# Uncertainty Principle and Power Quality Sensing and Analysis in Smart Substation

**DOI:** 10.3390/s20154281

**Published:** 2020-07-31

**Authors:** Wanli Cai, Li Wu, Yibo Cui, Shunfan He

**Affiliations:** 1 State Grid Hubei Electric Power Research Institute, Wuhan 430000, China; yanlang0304@126.com (W.C.); pt-5@163.com (Y.C.); 2 State Grid Wuhan Electric Power Supply CO., LTD., Wuhan 430000, China; wulee0913@sohu.com; 3 Collage of Computer Science, South-Central University for Nationalities, Wuhan 430000, China

**Keywords:** ideal atomic decomposition, power quality, uncertainty principle, waveform analysis

## Abstract

Different kinds of power quality can be sensed in a smart substation. Power quality sensing and analysis are basic functions of a smart substation for situation awareness. The uncertainty principle, which states that the time uncertainty and frequency uncertainty cannot be minimized simultaneously, is a bottleneck problem that undermines the faithfulness of sensing and confines the accuracy of analysis. This paper studies the influence of the uncertainty principle on the power quality monitoring issue in detail and solves the problem by ideal atomic decomposition (IAD). The new method employs a pair of time and frequency bases where the power quality waveform is sensed. Then, both time uncertainty and frequency uncertainty can be minimized simultaneously. The sensing process is realized by orthogonal matching pursuit (OMP). By simulated and field power quality tests with comparisons of developed methods, the new method can give faithful sensing and accurate analysis for various power qualities, and is validated as an effective power quality monitoring method in smart substations.

## 1. Introduction

Power substations play a key role in power transmission and distribution. A smart substation is typically implemented with a sophisticated combination of smart primary high-voltage equipment and hierarchically networked secondary devices. Based on the IEC 61850 communication protocol, the functionalities, such as the information sharing and interoperability among smart electric equipment, are realized in smart substations. They are regarded as the basis for the development of the smart grid and represents the future development trend of substation technologies [1]. Situation awareness is a new characteristic of the smart grid [2,3]. Power quality (PQ) analysis is usually used to evaluate the power quality level in traditional power substations. However, with smart electric equipment, power equipment failures, special loads, and some grid operations can also be viewed from the PQ waveform in smart substations. For example, a power cable fault can become known by the arc voltage waveform [4,5]; high-power DC loads, which need rectification by thyristors, can be monitored by the voltage notch [6]; and reactive power compensation by capacitor banks can be seen by voltage oscillation [7]. In all, PQ sensing and analysis cannot only be for PQ evaluation but also for smart grid situation awareness. Therefore, the influence of the uncertainty principle on PQ sensing and analysis requires more concern than ever before.

One big difference of PQ sensing and analysis in substations is that multiple kinds of power quality waveforms can be possibly measured. The field PQ can be in standard form, as described in [8,9], and can also be in irregular and complex forms. Hence, the main challenges of PQ sensing and analysis in the smart substation are:

1. It is unknown how many kinds of PQ can be sensed.
2. It is unknown what kinds of PQ are sensed.
3. Accurate features of PQ are needed to study what causes the PQ.

Feature sensing of PQ is the foundation of PQ analysis and key to face these challenges. As there are many methods for PQ sensing and each method has several improved versions, the classification of existing methods, the representative methods of each class, and the advantages and disadvantages of the methods are given in Table 1.

In this paper, ideal atomic decomposition (IAD) [20] is proposed to sense and analyze the PQ. IAD solves the disadvantage of the linear methods of non-parameter estimation thoroughly and also has each advantage of the methods. The highlighted contribution of the work in both the sensing of PQ time features and frequency features is faithful at the same time without prior knowledge of the PQ. The features of PQ have specific physical meaning and are faithfully sensed, which are competitive for facing the challenges of PQ monitoring in the smart substation.

The paper is organized as follows: Section 2 illustrates the influence of the uncertainty principle on PQ sensing and analysis; Section 3 proposes the IAD to obtain both accurate time and frequency features of the PQ; Section 4 shows the performance of the new method by simulated PQ tests; Section 5 gives cases of PQ sensing and analysis in a smart substation; Section 6 summarizes the whole work

## 2. Influence of Uncertainty Principle on Power Quality Sensing and Analysis

### 2.1. Uncertainty Principle of Power Quality Sensing

The power quality waveform is ultimately sensed by an analog-to-digital converter (ADC). Supposing the quantization number of the ADC is certain, the uncertainty in PQ sensing includes time uncertainty ($u_{t}$), which is controlled by sampling frequency (*F_s_*), and frequency uncertainty ($u_{f}$), which is controlled by sampling time (*T_s_*). If Min (.) returns the minimized value of “.”, then:

(1) $$\mathrm{Min}(u_{t})=1/F_{s},\mathrm{Min}(u_{f})=1/T_{s}$$

Reducing the uncertainty is the premise of faithful PQ sensing. However, regardless of how *F_s_* and *T_s_* are increased, there is

(2) $$u_{t}\times u_{f}\geq \frac{1}{4\pi }$$

because of the uncertainty principle [21]. Equation (2) is the bottleneck problem that undermines the faithfulness of sensing and confines the accuracy of analysis.

### 2.2. Uncertainty Principle and Accuracy of Power Quality Analysis in Substation

The kind of PQ is generally classified by its time–frequency features. Irregular and complex PQ can be deemed as a combination of the standard ones described in [8,9]. When facing Challenge 1 and Challenge 2, it is unknown how many kinds of PQ and what kinds of PQ will be sensed in the substation. In this situation, both time features and frequency features of the PQ are supposed to be sensed faithfully, or Challenge 3 will hardly be tackled. According to [8,9], there are three main kinds of PQ: Steady PQ (harmonics and interharmonics), transient PQ (impulse and oscillation), and short-duration PQ (sag, swell, and interruption), which are denoted as $pq_{1}(t)$, $pq_{2}(t)$, and $pq_{3}(t)$, respectively. Then,

(3) $$\left\{ \begin{array}{l} pq_{1}(t)=\sum_{k=1}^{n1}a_{k}\cos(\omega_{k}t+\theta_{k})\;\\ pq_{2}(t)=\sum_{k=1}^{n2}b_{k}(t)\delta (t-t_{k}),\;b_{k}(t)=b_{k}e^{-Tt}\;or\;b_{k}(t)=b_{k}cos(\omega_{o}t)e^{-Tt}\;\\ pq_{3}(t)=d\cos(\omega_{n}t)(\varepsilon (t-t_{1})-\varepsilon (t-t_{2})),\;\varepsilon (t)=\int_{-\infty }^{t}\delta (\tau )d\tau \end{array}\right.$$

where $a_{k}$, $\omega_{k}$, and $\theta_{k}$ are the amplitude, frequency, and phase of the *k*-th cosine component, respectively; *b_k_* and *t_k_* are the amplitude and time features of δ(t), respectively; *T* and $\omega_{o}$ are the decaying time and oscillatory frequency, respectively; *d*, *t*_1_, and *t*_2_ are the amplitude, start time, and end time of short-duration disturbances, respectively; $\omega_{n}$ is the fundamental frequency; and *n*1 and *n*2 are two positive integers. Then, an unknown PQ sensed in the substation can be written as:

(4) $$PQ=pq_{1}(t)+pq_{1}(t)+pq_{3}(t)+noise$$

**Figure 1:**
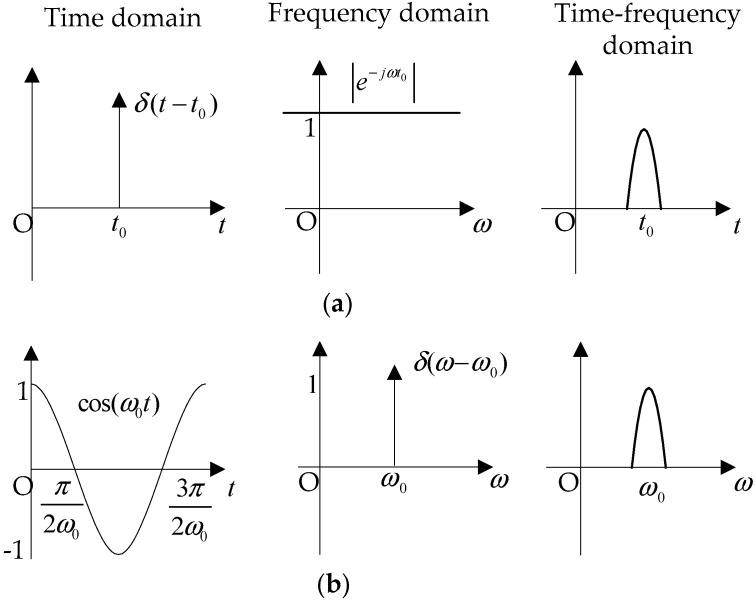
PQ signals sensed in the time domain, frequency domain, and time–frequency domain: (**a**) Transient PQ sensing; (**b**) steady PQ sensing.

The accuracy of PQ analysis mainly relies on the faithfulness of PQ sensing. Figure 1 is used to explain the relation of the sensing faithfulness and the analysis accuracy.

In Figure 1a, features (time index, amplitude, and polarity) of transient PQ can be analyzed accurately in the time domain and inaccurately in the time–frequency domain, while they cannot be analyzed in the frequency domain. In addition, in Figure 1b, features (amplitude, frequency, and phase) of steady PQ can be analyzed accurately in the frequency domain but inaccurately in the time–frequency domain, while they cannot be analyzed in the time domain. In Figure 1, if $u_{t}$ is minimized, transient PQ analysis will be accurate, but $u_{f}$ will be unacceptable and steady PQ analysis will not be done. On the other hand, if $u_{f}$ is minimized, steady PQ analysis will be accurate, but $u_{t}$ will be unacceptable and transient PQ analysis will not be done. In the condition where it is unknown how many kinds of PQ and what kinds of PQ will be sensed, the PQ is sensed in the time–frequency domain for balanced PQ analysis, similar to many developed methods. Although both transient PQ and steady PQ can be sensed in the time–frequency domain but neither of them is faithful, both analysis results of time features and frequency features are inaccurate. Therefore, the realization of Equation (1) is the premise to face the challenges of PQ sensing and analysis.

## 3. Ideal Atomic Decomposition by Orthogonal Matching Pursuit

### 3.1. Ideal Atomic Decomposition of Time and Frequency

Unlike traditional methods, which sense PQ in a single basis or domain, the IAD senses PQ in a pair of time and frequency bases. The key to realize Equation (1) simultaneously is to sense the transient PQ in the time basis only and to sense the steady PQ in the frequency basis only. Suppose **x** is an *n*-dimensional-sampled PQ signal, and the **x** sensing in basis **A** and the sensed coefficients γ can be written as:

(5) x=Aγ

In traditional methods, **A** is an *n* by *n*-dimensional matrix such as a Fourier matrix, Gabor matrix, and wavelet matrix, and γ is an *n*-dimensional-sensed coefficient vector of the corresponding matrix. Denote the time basis (**I**) and frequency basis (**F**) as:

(6) $$I={\left(\begin{array}{llll} 1 & 0 & \ldots & 0\\ 0 & 1 & \ldots & 0\\ \vdots & \vdots & \ddots & \vdots \\ 0 & 0 & \ldots & 1 \end{array}\right)}^{n\times n},F=\sqrt{\frac{1}{n}}{\left(\begin{array}{cccc} 1 & 1 & \ldots & 1\\ 1 & e^{-j2\pi /n} & \ldots & e^{-j2\pi (n-1)/n}\\ \vdots & \vdots & \ddots & \vdots \\ 1 & e^{-j2\pi (n-1)/n} & \ldots & e^{-j2\pi {\left(n-1\right)}^{2}/n} \end{array}\right)}^{n\times n}.$$

Then, the **x** sensing in the pair of bases can be expressed as:

(7) $$x=A\gamma =[I,F]\left[\begin{array}{l} \gamma_{I}\\ \gamma_{F} \end{array}\right]$$

where $\gamma_{I}$ and $\gamma_{F}$ are the coefficient vectors of **I** and **F**, respectively.

### 3.2. Realization of Ideal Atomic Decomposition by Orthogonal Matching Pursuit for Power Quailty Sensing

In Equation (7), **A** is an *n* by 2*n*-dimensional matrix, and γ is a 2*n*-dimensional vector. Equation (7) is a group of underdetermined linear equations. The solution of γ can be expressed as:

(8) $$\mathrm{Min}{\left\|\gamma \right\|}_{0}\;subject\;to\;x=A\gamma$$

where ${\left\|.\right\|}_{p}\;$ returns the *p* norm of “.”. The solution of Equation (8) is a non-deterministic polynomial hard, which is approximated as:

(9) $$\mathrm{Min}{\left\|\gamma \right\|}_{1}\;subject\;to\;x=A\gamma$$

The solution of Equation (9) can be realized by orthogonal matching pursuit (OMP) [22] and works as:

Symbol description: $re_{t}$, $\Lambda_{t}$, and $\gamma_{t}$ are the error vector, index set, and solution vector, respectively, after *t* times iteration; ∅ is the empty set; $\lambda_{t}$ is the index that is found at the *i*th iteration; $\alpha_{j}$ is the *j*th vector of **A**; and **A***_t_* is a vector set that is selected from **A** according to $\Lambda_{t}$.

Step 1. Initialize $re_{0}=x$, $\Lambda_{0}=\emptyset$, $A_{0}=\emptyset$, *t* = 1;

Step 2. Find the index of the maximum inner product of $re_{t-1}^{}$ and $\alpha_{j}$:

(10) $$\lambda_{t}=\arg(\underset{i=1,\ldots ,n}{\max}\left|re_{t-1}^{T},\alpha_{i}\right|)$$

Step 3. Refresh $\Lambda_{t}$ and **A***_t_*, $\Lambda_{t}=\Lambda_{t}\cup \lambda_{t}$ and $\;A_{t}=A_{t}\cup \alpha_{\lambda_{t}}$;

Step 4. Find the least squares solution of $x=A_{t}\gamma_{t}$:

(11) $${\hat{\gamma }}_{t}=\arg(\underset{r_{t}}{\min}\left\|x-A_{t}\gamma_{t}\right\|)={\left(A_{t}^{T}A_{t}\right)}^{-1}A_{t}^{T}x$$

Step 5. Refresh the error vector; then, *t = t +* 1:

(12) $$re_{t}=x-A_{t}{\hat{\gamma }}_{t}=x-A_{t}{\left(A_{t}^{T}A_{t}\right)}^{-1}A_{t}^{T}x$$

Step 6. Finish the **A** representation. If $\left|re_{t}\right|$ is less than the threshold *Th*, then the demanded recovered accuracy is satisfied and the iteration stops. The indexes and coefficients of the selected $\alpha_{j}$ for PQ representation in **A** can be found in $\Lambda_{t}$ and $\gamma_{t}$, respectively.

In Equation (10), at each iteration, OMP tries to find the signal component with the largest energy in **x** by matching it with the atom $\alpha_{j}$ of **A**. Because **F** is the frequency matrix, the steady PQ will be matched by the $\alpha_{j}$ of **F**. In addition, because **I** is a time matrix, the transient PQ will be matched by the $\alpha_{j}$ of **I**. In Equation (11), when the $\alpha_{j}$ is found, the $\gamma_{j}$ is calculated by the least squares method. In Equation (12), the remaining signal is obtained by removing the signal that has been sensed by the $\alpha_{j}$s of the **A,** and when the $\left|re_{t}\right|$ is less than the threshold, all signals larger than the threshold in PQ are sensed.

### 3.3. Power Quality Analysis Based on Ideal Atomic Decomposition Sensing

The typical signal-to-noise ratio (SNR) of **x** is 40 dB [8], and the $\left|re_{t}\right|$ is set to 1%. The PQ analysis is based on the sensed coefficient γ of IAD.

#### 3.3.1. Transient Power Quality Analysis

**Figure 2:**
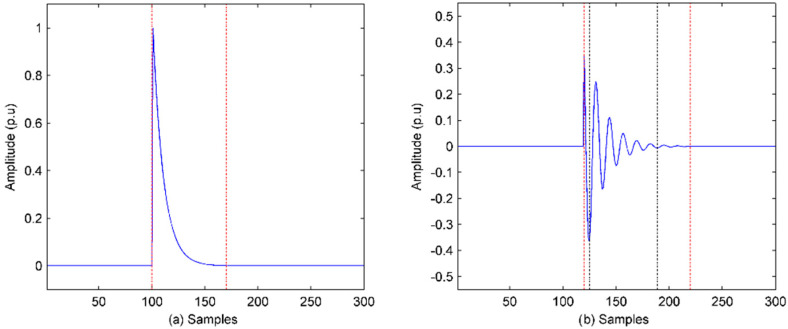
Transient PQ: (**a**) Transient impulse; (**b**) transient oscillation.

Transient PQ is sensed in **I** and the features can become known from $\gamma_{I}$. The transient impulse and transient oscillation are shown in Figure 2a,b, respectively.

For impulse analysis, in Figure 2a, denote $\lambda_{1}$ as the index of the first nonzero $\gamma_{I}$ and $\lambda_{2}$ as the index of the last nonzero $\gamma_{I}$; the amplitude is the largest value of the nonzero $\gamma_{I}$. The signs of each nonzero $\gamma_{I}$ are the same and indicate the polarity of the impulse. The duration of impulse is:

(13) $$D=(\lambda_{1}-\lambda_{2})/F_{s}$$

For oscillation analysis, in Figure 2b, the start time, duration, and amplitude are evaluated as the same as those of impulse. The oscillatory frequency is calculated as:

Step 1. Find the extreme points. The smallest amplitude of the points is more than 0.01 p.u.

Step 2. Choose the second extreme point and denote it as *ep_1_*. Then, find all the extreme points that share the same sign with *ep_1_* and **EP** = [ *ep_1_*, *ep_1_*,…,*ep_k_*]. As shown in Figure 2b, the first *ep* and last *ep* are indicated by the two black dashed lines.

Step 3. Denote the time duration *D_e_* of *ep_1_* and *ep_k_*; the oscillatory frequency is:

(14) $$f_{o}=(k-1)/D_{e}$$

#### 3.3.2. Short-Duration Power Quality Analysis

Features of short-duration PQ are mainly obtained from the root-mean-square (RMS) value of the fundamental component of **x**.

Suppose that rms(.) returns the RMS of “.” and $\gamma_{f}$ is the fundamental group of $\gamma_{F}$ [9]; then, the fundamental component **x***_f_* in the time domain is

(15) $$x_{f}=F\gamma_{f}$$

The start time and duration can be known when the rms(**x***_f_*) crosses the threshold, which is 0.9, 1.1, and 0.1 p.u for sag, swell, and interruption, respectively.

#### 3.3.3. Steady Power Quality Analysis

Steady PQ is sensed in **I** and the features can become known from $\gamma_{F}$. Suppose that Re(.) and Im(.) return the real value and imaginary value of “.,” respectively; $\lambda_{j}$ is the *j*-th index of nonzero $\gamma_{F}$, and $\gamma_{j}$ belongs to $\gamma_{F}$. The amplitude (*a*), frequency (*f*), and phase (*p*) of the *j-*th steady PQ can be respectively calculated as:

(16) {aj=Re2(γj)+Im2(γj)fj=λjTspj=tan−1(Im(γj)Re(γj))

By the IAD method, some rules can be designed to simplify the PQ analysis.

Rule 1. If $\gamma_{I}\neq 0$, there is transient PQ and R1 = 1, or R1 = 0 and there is no transient PQ.

Rule 2. If R1 = 1, and if the polarities of nonzero $\gamma_{j}\in \gamma_{I}$ remain the same, the transient PQ is impulse and R2 = 1. Then, amplitude and polarity are obtained, and the duration is calculated as Equation (13). If R1 = 1, and if the polarities of nonzero $\gamma_{j}\in \gamma_{I}$ are not the same, the transient PQ is oscillation and R2 = 0. Then, amplitude, polarity, and duration are obtained as the same as impulse, and the oscillatory frequency is calculated as Equation (14).

Rule 3. If the rms(**x***_f_*) crosses the threshold, then R3 = 1, and according to the threshold type, cross-points of rms(**x***_f_*), and the extreme point of rms(**x***_f_*), the type, start time, duration, and disturbed RMS can become known. Or, R3 = 0 and there is no short-duration PQ.

Rule 4. If $\gamma_{F}\neq 0$ except for $\gamma_{f}$, there are steady PQs, and R4 = 1. The features are calculated as Equation (15).

Rule 5. If the nonzero $f_{j}$ is integer times the fundamental frequency, there is harmonic and R5 = 1. If not, there is an interharmonic and R5 = 0.

**Figure 3:**
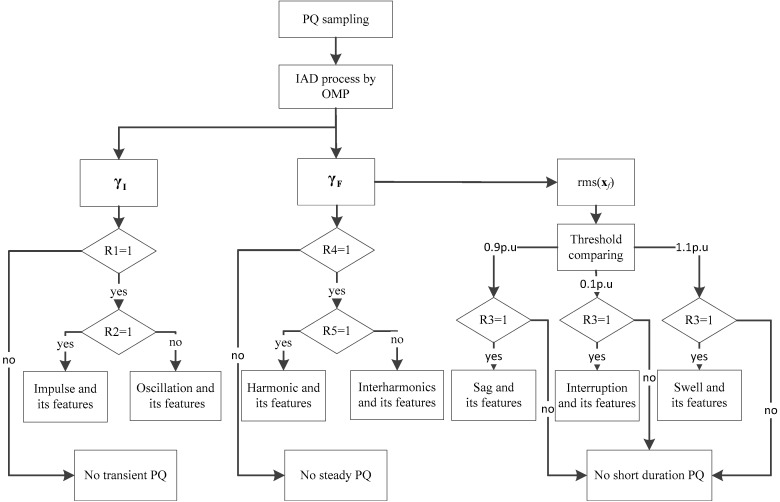
Scheme of PQ sensing and analysis by the new method.

Then, the scheme of PQ sensing and analysis is as shown as Figure 3. The main differences of the IAD and traditional methods are:

1. IAD senses the PQ in a pair of time and frequency bases, and Equation (1) can be realized, which can hardly be done by many traditional methods because of Equation (2). Consequently, with minimized $u_{t}$ and $u_{f}$, PQ sensing by IAD is more faithful than traditional methods.
2. Each of the PQ feature sensing by IAD strictly complies with the definitions of PQ in [8,9], and thus has specific physical meaning. Consequently, with all these explainable and sensed features, the event of PQ has chances to be revealed.

## 4. Simulation of Typical Power Quality Sensing and Analysis

In this section, transient, steady, and short-duration PQs are sensed and analyzed by IAD with comparisons of developed methods. The influence of the uncertainty principle on traditional methods and the performance of IAD against the influence are also presented in detail. According to [8], the *Fs* is 6400 Hz and the SNR is 40 dB without specification (noise magnitude is 0.01 p.u). Hence, the threshold $\left|re_{t}\right|$ is 0.01 p.u, which means samples whose amplitudes are smaller than 0.01 p.u will not be sensed by IAD.

### 4.1. Transient and Steady Power Quality Test

**Figure 4:**
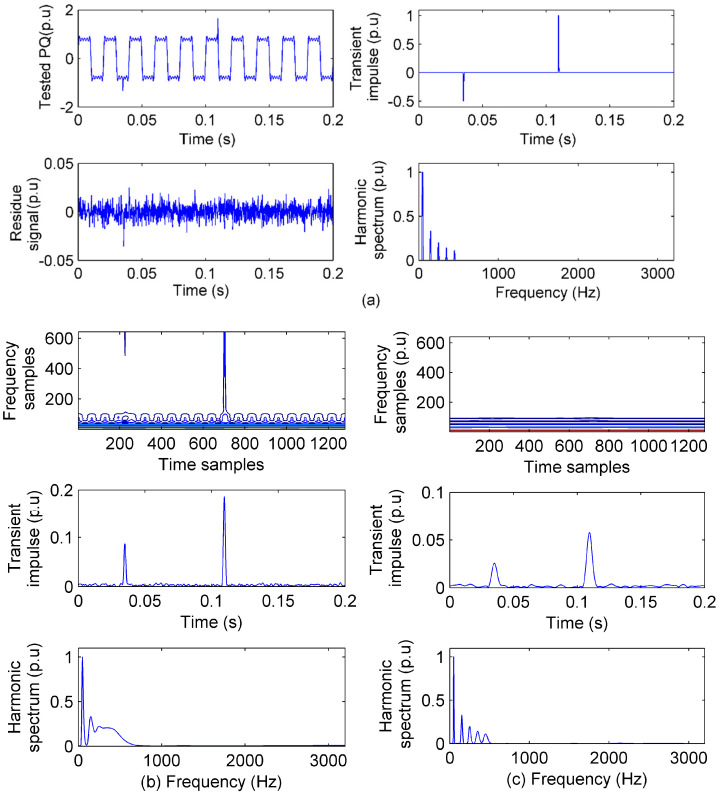
Transient and steady PQ sensing with 40 dB noise: (**a**) The PQ signal, residue signal, transients sensed in time, and spectrum of harmonics sensed in frequency by ideal atomic decomposition (IAD); (**b**) contour of PQ, and the transients and harmonics sensed by the S transform when *u_t_* = *u_f_*; (**c**) contour of PQ, and the transients and harmonics sensed by the S transform when *u_t_* > *u_f_*.

A PQ with transient impulses and steady harmonics is simulated as Equation (17) whose sensing from IAD and the S transform are shown in Figure 4. The S transform is a widely used PQ analysis method in time–frequency. The S transform has a Gaussian window, which is controlled by a factor for either better time resolution (smaller *u_t_*) or better frequency resolution (smaller *u_f_*) [23].

(17) $$\left\{ \begin{array}{l} \mathrm{impulses}:x_{1}=e^{-6400(t-0.11)},\;x_{2}=0.5\;e^{-3200(t-0.035)}\\ \mathrm{harmonics}:x_{3}=\sum_{i=1}^{9}\frac{1}{i}\sin(2\pi i50t),\;(i\;\mathrm{is}\;\mathrm{odd}\;\mathrm{number})\;\\ x=x_{1}+x_{2}+x_{3}. \end{array}\right.$$

In Figure 4a, it can be seen clearly that the impulses are faithfully sensed by $\gamma_{I}$ and the harmonics are faithfully sensed by $\gamma_{F}$. The $\left|re_{t}\right|$ of the residue signal, which is mainly from the noise, is within 0.01 p.u. Then, with the faithful PQ sensing, the features are obtained and shown in Table 2.

In Figure 4b, the PQ sensed by the S transform is shown when the factor of the Gaussian window is 1 (*u_t_* = *u_f_*) [23], including the contour of the matrix of the S transform and the sensing of impulses and harmonics from the contour. Obviously, the sensing of transient impulses is unfaithful as the impulses are expanded. Besides, the sensing of harmonics is also unfaithful as the spectrum becomes a successive curve rather than discrete impulses.

In Figure 4c, in order to sense the more faithful harmonics, the Gaussian window factor of the S transform is set to 5, which reduces the *u_f_*. The spectrum of harmonics becomes more discrete than that in Figure 4b; however, the transient impulses sensing expands more because of Equation (2). When *u_f_* reduces, *u_t_* increases, and the transient impulses sensing becomes more unfaithful.

In Figure 4, the influence of the uncertainty principle on PQ sensing and analysis by the S transform is illustrated, and the results of PQ analysis when *u_t_* = *u_f_* (ST1) and *u_t_* > *u_f_* (ST2) are also presented in Table 2. The sensing performance and analysis results demonstrate that regardless of how the Gaussian window is improved, the balanced analysis is always obtained because of the uncertainty principle. On the contrary, only transient impulses are sensed in the time basis and only steady harmonics are sensed in frequency by IAD; the PQ sensing is faithful and the analysis results are precise within the accuracy of $\left|re_{t}\right|$.

### 4.2. Short-Duration Power Quality Test

Short-duration PQ includes voltage sag, swell, and interruption, which are evaluated by RMS. As the single-phase fault is the most common fault on the grid, a voltage sag signal is simulated as Equation (18).

(18) $$x=\left\{ \begin{array}{l} 0.5\sin(2\pi 50t),\;0.065s<t<0.11s\\ \sin(2\pi 50t)\; \end{array}\right.$$

**Figure 5:**
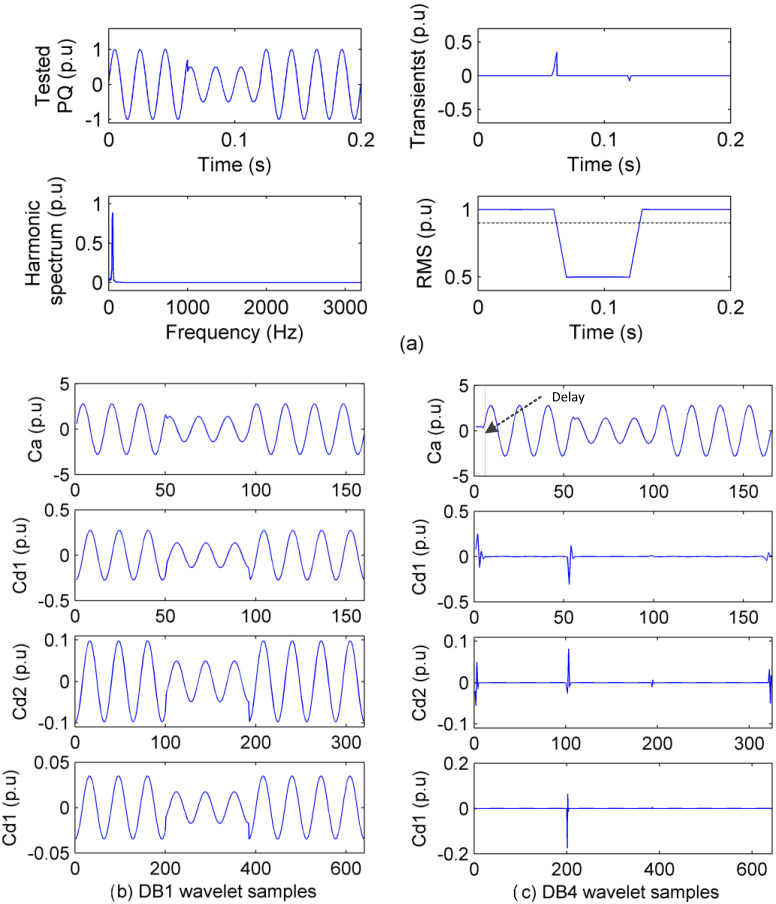
Voltage sag sensing with 40 dB noise: (**a**) The sag signal, the sensing of transients in time, the sensing of the fundamental component in frequency, and the RMS of voltage sag by IAD; (**b**) wavelet coefficients from voltage sag sensing by DWT with DB-1 wavelet basis; (**c**) wavelet coefficients from voltage sag sensing by DWT with DB-4 wavelet basis.

In many analysis cases, the transients of short-duration PQ are also important; therefore, discrete wavelet transform (DWTs) are usually used for short-duration PQ analysis [24]. In this case, DWTs are employed as the comparison. In addition, to show the uncertainty principle influence on the DWT, two kinds of wavelet bases, Daubechies-1 (DB-1) wavelet basis (DWT1) and DB-4 wavelet basis (DWT2), which is longer than the DB-1 wavelet basis, are used. In the figure illustration, Ca, Cd3, Cd2, and Cd1 are the approximation coefficient, detail 3 level coefficient, detail 2 level coefficient, and detail 1 level coefficient, respectively. The wavelet transform has border distortion, which causes sensing delay. A longer wavelet basis brings worse border distortion and longer delay [24]. To minimize the distortion and reduce the delay, the raw PQ waveform has to be lengthened by zeros at the two borders. In this case, we just use the original PQ waveform for DWT analysis. The sensing performance of IAD, DWT1, and DWT2 is shown in Figure 5 in detail and the analysis results are presented in Table 3.

In Figure 5a, sudden changes in voltage sag are sensed faithfully by $\gamma_{I}$. In addition, the disturbed fundamental component is sensed faithfully by $\gamma_{F}$. Then, the fundamental component recovered by Equation (15) compared to the threshold is obtained.

In Figure 5b, Ca senses the shape of the original PQ faithfully, with obvious energy leakage of approximation coefficients to detailed level coefficients, however. Consequently, the measurement of sag depth will be smaller than the true one.

In Figure 5c, the energy leakage is reduced by a longer wavelet basis; however, each level coefficient is delayed. For example, the Ca is distorted at the start border and delayed by six approximation samples (about 7 ms) compared to that in Figure 5b. Consequently, the whole waveform analysis is delayed.

In Figure 5, the longer wavelet basis brings less energy leakage but worse border distortion and longer time delay, while a shorter wavelet basis brings more energy leakage but less border distortion and less time delay. Consequently, in Table 3, the analysis results by DWT are inaccurate in either sag depth or start time. Adding zeros to the raw PQ waveform at the two borders may solve the problem but with the premise that the wavelet basis is certain. However, one certain wavelet basis can hardly be suitable for multiple kinds of PQs, and the number of zeros that should be added changes case by case. On the contrary, the IAD can give accurate analysis, as shown in Table 3, and no parameter or pre-setting changes of the method, because IAD senses the PQ in a pair of time and frequency bases, and both *u_t_* and *u_f_* are minimized simultaneously. Then, different kinds of PQs are sensed in different bases separately.

### 4.3. Power Quality with Strong Noise Test

In real PQ monitoring, the noise magnitude is within 0.01 p.u (SNR > 40 dB) typically [8]. However, the noise can increase if there is strong electromagnetic interference. In 4.1 and 4.2, the noise whose magnitude is 0.01 p.u has been tested, and the stronger noise influence on PQ analysis is tested in this case. Because time features and frequency features are most concerned in PQ analysis, Equation (17) is employed but its SNR is changed to 30 dB (noise magnitude is 0.0316 p.u) and the threshold is still 0.01 p.u.

**Figure 6:**
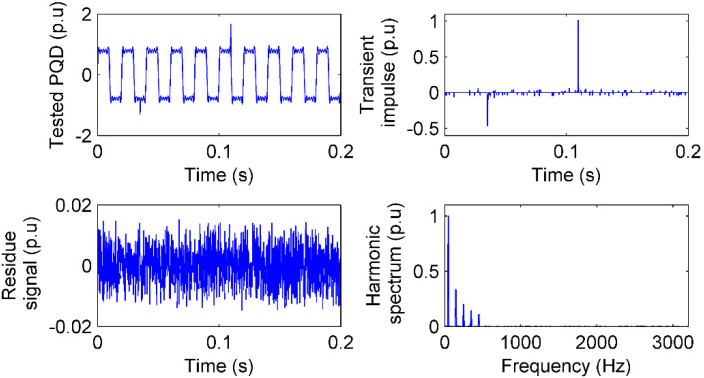
Transient and steady PQ sensing with 30 dB noise: The PQ signal, residue signal, transients sensed in time, and spectrum of harmonics sensed in frequency by IAD.

In Figure 6, the tested PQ signal, residue signal, transient impulse sensing, and harmonic sensing are presented. As the residue signal shows, the difference in the sensed PQ and the real PQ is still retained by the threshold. With the strong noise, sensing of steady PQ is affected little while that of transient PQ is not. The reason is that the noise is more like a random successive transient disturbance rather than a periodic one. However, even with strong noise contamination, the sensing of both transient PQ and steady PQ are still clear compared to those in Figure 4.

### 4.4. Flicker and Interharmonics PQ Test

**Figure 7:**
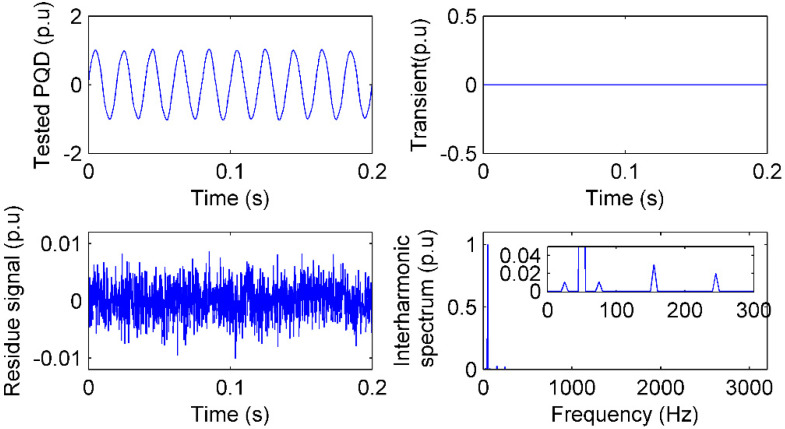
Flicker and interharmonic test with 40 dB noise: The PQ signal, residue signal, transients sensed in time, and spectrum of flicker and interharmonics sensed in frequency by IAD.

Flicker is a lamp maloperation caused by voltage fluctuation, which is a special kind of interharmonic [25]. Following [25], in this case, a flicker with interharmonics is simulated as:

(19) x=(1+0.02sin(2π25))sin(2π50)+0.03sin(2π155)+0.02sin(2π245)

and the IAD sensing is given in Figure 7.

Because there is no transient PQ in Equation (19), the $\gamma_{I}$ is 0 in Figure 7. In the spectrum, two 0.01 p.u interharmonics at 25 and 75 Hz can be found in the spectrum, which result in the flicker. A 0.03 p.u interharmonic is sensed at 155 Hz and a 0.02 p.u interharmonic is sensed at 245 Hz. The PQ sensing is faithful to Equation (19) and the features of PQ are accurate, consequently.

### 4.5. Notch Test

Voltage notch is a periodic voltage disturbance caused by the normal operation of power electronics devices [26]. The notch depth and notch area are concerned features to show the influence of power electronics devices, especially the three-phase silicon-controlled rectifier, on power quality. A notch signal is simulated as:

(20) $$\begin{array}{ll} x_{1}=0.3\;(\varepsilon (t-t_{1})\;-\varepsilon (t-t_{2})\;) & \left(\;t_{1}=i\times 0.02s,t_{1}-t_{2}=2.5ms,i=1,2,\ldots ,9\right)\\ x_{2}=\cos(2\pi 50t), & x=x_{1}-\;x_{2}\; \end{array}$$

**Figure 8:**
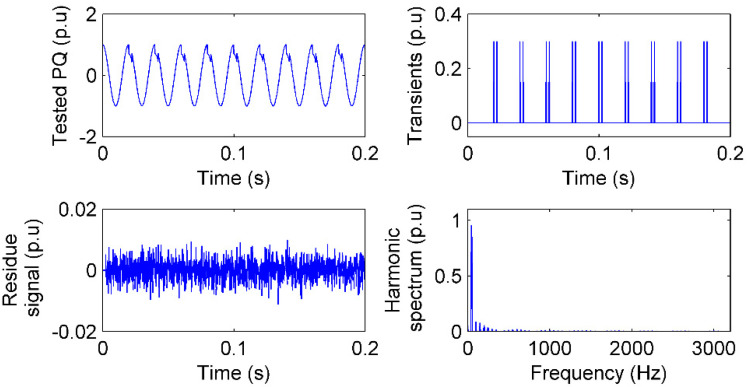
Notch test with 40 dB noise: The PQ signal, residue signal, transients sensed in time, and spectrum of harmonics sensed in frequency by IAD.

The notch depth is 0.3 p.u and the notch area is at the peak of each cycle with 2.5 ms duration. The notch sensing by IAD is given in Figure 8.

The notch is one of the typical time–frequency PQ signals. Changes in the notch are a transient PQ and the duration of the notch is a square waveform, which can be deemed as a composition of odd-order harmonics. In Figure 8, the start and end of each notch are sensed faithfully in **I**, the transient amplitude is 0.03 p.u corresponding to the notch depth, and the time difference of the start and end is 2.5 ms. In addition, odd harmonics from the 3rd to 21st are sensed in the spectrum, which means that there is a square waveform, and the fundamental component is smaller than 1 p.u, which means the fundamental component is reduced by the square waveform. The PQ is then identified as a notch.

In these cases, it should be emphasized that the IAD cannot only classify the type of PQ, but also give faithful sensing of PQ in time and frequency simultaneously, where it is much more important and valuable to discover the event of PQ, because different events can result in the same type of PQ but with different time–frequency features whose revealing needs faithful sensing of PQ in time and frequency simultaneously.

## 5. Field Power Quality Sensing and Analysis in Smart Substation

**Figure 9:**
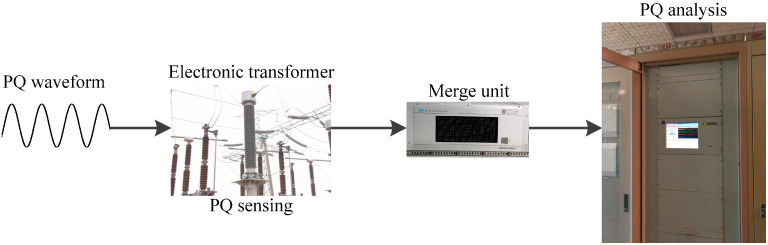
Power quality sensing system in 220 kV power substation.

A scheme of a power quality sensing and analysis system in a 220 kV smart substation, Huangang, Hubei province, China is shown in Figure 9. Electronic transformers whose errors are within 0.2% are used to measure and sample the power quality disturbances, and the quantization is 16 bits. In this step, time uncertainty and frequency uncertainty are brought in depending on the sampling rate and sampling time. According to IEEE 1159-2009 [8], the sampling rate is set as 6.4 kHz (128 samples per cycle). For three-phase power quality monitoring, the digital power quality waveforms are collected in the merge unit with time labels. Then, according to IEC 61850-9-1 [27], 100 M bits-per-second fiber communication is used to send the signal from the measurement system to an industrial computer (CPU intel i9 with 8 cores at 3.6 GHz and 32 GB random access memory at 2.6 GHz). The new method runs in the industrial computer for PQ analysis. As the typical noise magnitude of power quality sensing is within 0.01 p.u, the $\left|re_{t}\right|$ is set as 0.01 p.u.

In the following different case studies, it should be highlighted that the IAD has no pre-setting changes and faces the challenges of PQ sensing and analysis in the smart substation directly with the simultaneously minimized *u_t_* and *u_f_*.

### 5.1. Events of Voltage Sag Analysis

**Figure 10:**
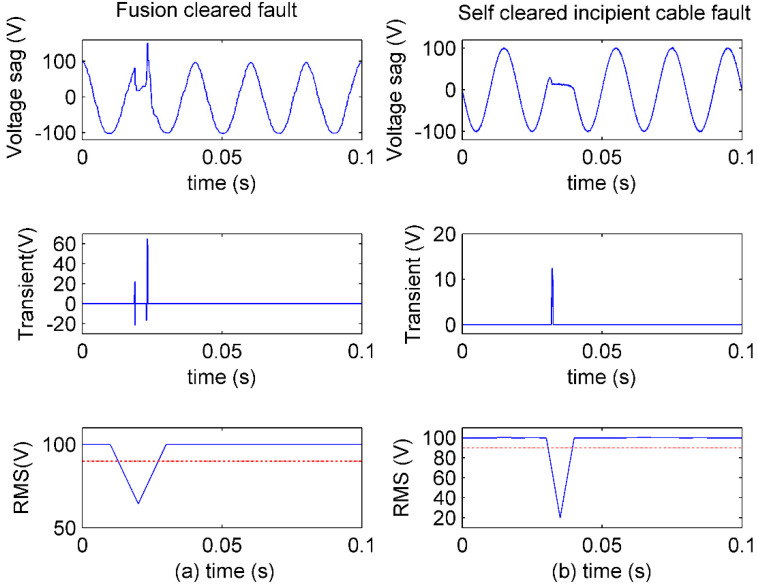
IAD performance of sub-cycle voltage sags sensing and analysis: (**a**) Voltage sag signal from fusion-cleared fault, the transients sensed in time, and the RMS evaluation; (**b**) voltage sag signal from incipient cable fault, the transients sensed in time, and the RMS evaluation.

Figure 10 shows two similar sub-cycle voltage sags caused by different events, which are sensed in the smart substation.

In Figure 10a, the voltage sag is caused by a fault, which is cleared by fusion blow. Fusion is often used for line protection from damages caused by large current. The voltage sag caused by large current can be very short, usually in sub-cycle duration, if there is fusion protection. However, the sudden change in large current will result in a large transient impulse when the fault is cleared (at the end of voltage sag). In Figure 10b, the voltage sag is caused by an incipient cable fault, which is self-cleared. The incipient cable fault generates arc voltage, whose phase is the same as that of arc current [28]. When the arc current crosses the zero point, the arc voltage distinguishes itself. Hence, there is no transient impulse at the end of sag. As the mechanisms of the two voltage sags suggest, sag depth, sag duration, and transient impulse at the end of voltage sag are necessary to identify the events of the sags.

In Figure 10a, there are two impulses at 18 and 23 ms. The voltage sag starts at 14 ms and ends at 26 ms by RMS, and the duration is within a cycle (20 ms). The sag depth is 64.5 V. It can be seen clearly that there is a large impulse near the end of voltage sag. Then, the fault cleared by fusion blow is identified.

In Figure 10b, there is only one impulse at 32 ms. The voltage sag starts at 31 ms and ends at 39 ms by RMS, and the duration is within a cycle (20 ms). The sag depth is 19.8 V. It can be seen clearly that there is only an impulse near the start of voltage sag and no transient at the end of voltage sag. Then, an incipient cable fault is discovered by the self-cleared sub-cycle voltage sag.

### 5.2. Event of Harmonic Analysis

Harmonics are a common PQ, which can be sensed in the substation. Different harmonics are caused by different events, which are characterized by the total harmonic distortion (THD) and specific order of the harmonic.

**Figure 11:**
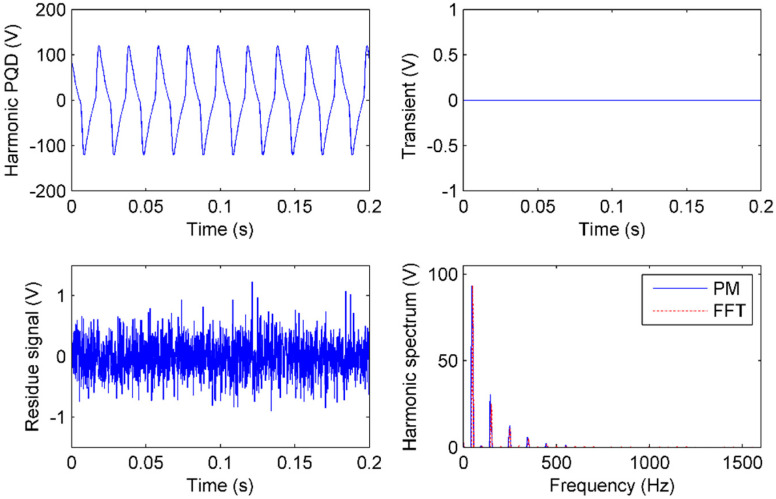
IAD and FFT performance of harmonic sensing: The harmonic signal, residue signal, transients sensing in time, and spectrum of harmonics in frequency by IAD and FFT.

The switch of an idle transformer online needs special control strategies to avoid deep core saturation. In this case, an intense harmonic is sensed from a maloperation of the idle wye-wye transformer switch. One remarkable part of the event is that the 3rd harmonic is extremely large [29]. The experimental results reveal that the 3rd harmonic is usually over 20% of the fundamental component. Figure 11 shows the harmonic signal and its sensing by IAD. In this case, we use fast Fourier transform (FFT) as the comparison because there are only harmonics.

In Figure 11, there are no transients in the harmonic, so the $\gamma_{I}$ is 0. The harmonic spectra of the IAD and FFT are the same, which verifies that all harmonic components are only sensed by $\gamma_{F}$. Both results of IAD and FFT show that there are 1st, 3rd, 5th, 7th, and 9th harmonics whose amplitudes are 93.4, 25.2, 11.0, 5.2, and 1.8 V respectively. As shown in Figure 3, the PQ type is harmonic. The total harmonic distortion (THD) is 30% and the 3rd harmonic is 27% of the fundamental component. With these evidences, a maloperation of the idle wye-wye transformer switch can be deduced.

### 5.3. Event of Multi-Oscillation Analysis

Transient oscillations are usually caused by a switch of capacitor banks. The typical duration is from half a cycle (10 ms) to several cycles and the oscillatory frequency is usually below 900 Hz. The vacuum interrupter of the capacitor banks will bear the recovery voltage after breaking the capacitive current. If the dielectric recovery strength of the vacuum gap cannot bear the recovery voltage, the vacuum interrupter will be stroked through [30]. A strike can occur several times and continuously in a short time, and the time interval is variable. The duration of the strike oscillation is less than half a cycle, and the oscillatory frequency is higher than the one of capacitor bank switches. In this case, multi-oscillations from the restrike of capacitor banks is used to test the IAD for field transient PQ sensing and analysis. Prony is used as the comparison with the condition that the location and number of oscillations are known [17].

**Figure 12:**
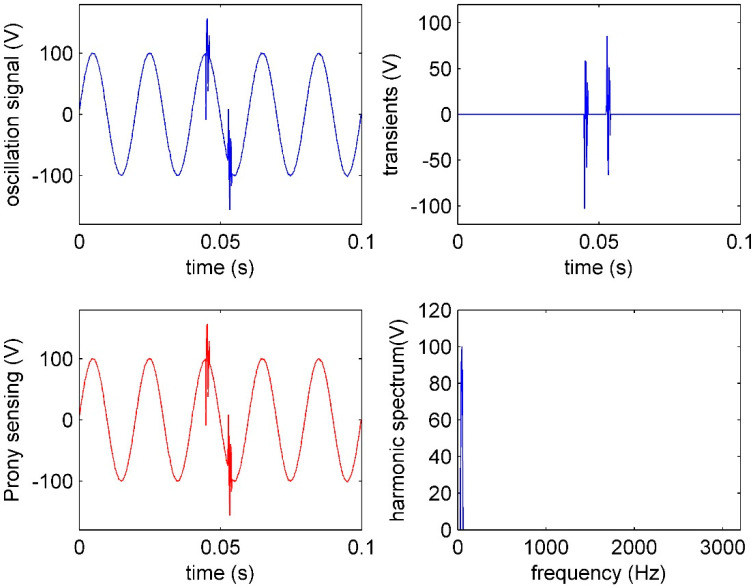
IAD and Prony performance of oscillation sensing: The oscillation signal, Prony trace of the oscillation, transients sensing in time, and spectrum of harmonics in frequency by IAD.

Figure 12 shows multi-oscillations sensed in the smart substation. The transients are sensed faithfully by $\gamma_{I}$, and only the fundamental component is in the spectrum. The multi-oscillations are sensed by IAD. The amplitudes of the two oscillations are 100.5 and 98.2 V, and the frequencies of the two oscillations are 1253 and 1248 Hz, respectively, which comply with the results of Prony analysis. The durations of the two oscillations are within half a cycle. The high frequency, large amplitude, short duration, and multi-occurrences of the oscillation suggest that the vacuum interrupter of capacitor banks is stroked through.

It should also be noted that the Prony method, as a parameter estimation method, gives an accurate analysis result because there is prior knowledge of the multi-oscillations, which has two oscillations and one sinusoidal component. If without the prior knowledge, the result of the parameter estimation method will not be right.

### 5.4. Event of Complex Power Quality Analysis

In a smart substation, complex power quality can also be sensed. The complex PQ can be deemed as a combination of several kinds of PQs. A permanent cable fault will result in arc voltage, which is characterized with a very low square shape voltage and transient impulses near the start of each half cycle. Hence, the arc voltage is one kind of typical complex PQ, which has both transient and steady disturbances.

Permanent cable fault will result in arc voltage, which is characterized as a very low square shape voltage and transient impulses near the start of each half cycle. Hence, the arc voltage is one kind of typical complex PQ that has both transient and steady PQs. Because of Equation (2), few time–frequency methods can minimize *u_t_* and *u_f_* at the same time, and sense the PQ faithfully (as discussed in the example of 3.1), and we use $\left|re_{t}\right|$ to reflect the analysis accuracy of IAD.

**Figure 13:**
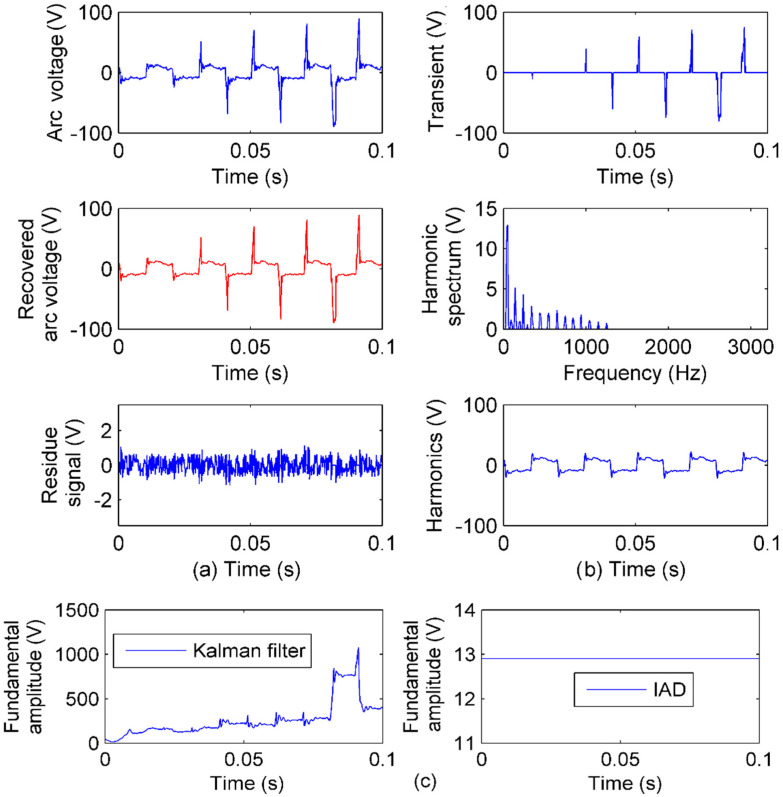
IAD performance of arc voltage sensing: (**a**) The arc voltage, recovered arc voltage by IAD sensing, and residue signal; (**b**) the transient sensing, the harmonic sensing in the frequency spectrum, and the recovered harmonic in time; (**c**) fundamental amplitude from Kalman filter and IAD.

Figure 13 shows the arc voltage sensed in the substation and IAD sensing performance. In Figure 13a, the recovered arc voltage is from faithful IAD sensing. In Figure 13b, the transients are sensed in $\gamma_{I}$. Compared to the arc voltage signal, the sensed transients represent the transients of the arc voltage faithfully. There are clearly impulses of about a half-cycle interval. The harmonics are sensed in $\gamma_{F}$. It can be seen that there are many odd harmonics which, together, make the signal like a square waveform. The representation of harmonics in time is derived from the $\gamma_{F}$ and also shown in Figure 13b. Together, the transients and harmonics in Figure 13b make the recovered arc voltage in Figure 13a, and the sensing accuracy is within 1% as shown by the residue signal.

Based on the faithful sensing of arc voltage by IAD, the PQ features are obtained following Figure 3: There are eight transient impulses, and the interval of seven impulses is about 0.01 s (half a cycle). There are intense harmonics mainly included from the 3rd to 25th odd harmonics, as well as small 2nd and 4th harmonics, and the RMS of the fundamental component is 12.9 V, which is near the threshold of interruption (10 V). All these evidences demonstrate that there is an arc voltage, and a cable is with permanent fault.

In the PQ analysis, the fundamental amplitude is always one of the most important features. As it has been proved that linear time–frequency transforms cannot give faithful PQ sensing of both time features and frequency features in Section 2 by theory and in Section 4 by experiments, and both time features and frequency features are important to reveal the event of arc voltage, a Kalman filter, as a representative method of parameter estimation, is compared in this case. The filter is modeled with 31-order for 15 different frequency components estimation. With higher order, the filter model will have risk at divergence [15,16].

In Figure 13c, the fundamental amplitude estimation is shown, and the amplitude obviously does not comply with the arc voltage. The reason is that the arc voltage has many frequency components (much more than 15 in the spectrum of Figure 13b). When there is a difference in measured signal and the model of parameter estimation, the result can hardly be accurate. On the contrary, prior knowledge of the number of signal components is not necessary for IAD, because of the minimization of both *u_t_* and *u_f_* at the same time.

### 5.5. Customer-Caused Failure of Irreuglar Power Quality Analysis

**Figure 14:**
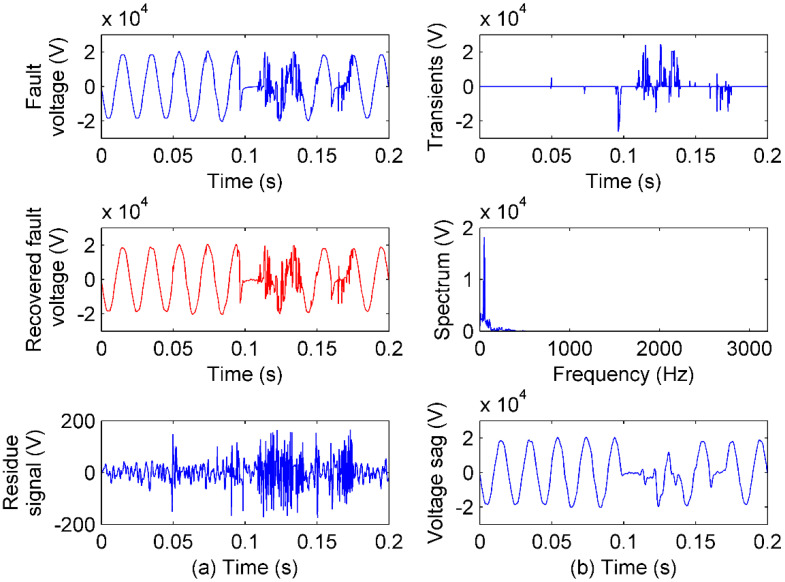
IAD performance of irregular PQ sensing: (**a**) The PQ signal, recovered PQ signal by IAD sensing, and residue signal; (**b**) the transient component sensing, the steady component sensing in the frequency spectrum, and the multi-sags in time.

In this case, an irregular PQ from customer-caused failure in a PQ disturbances library [31] is tested. With the validated advantages both in theory and experiments, the feature sensing by IAD is given in Figure 14.

In Figure 14, when intense transients are sensed by $\gamma_{I}$, the multi-sags sensed by $\gamma_{F}$ are displayed clearly. The sags in multi-stages show the multi-protections of the fault, and the transients show the fierce disturbances when the arc is extinguished and reignited. With these evidences, the customer may cause severe failure.

In this section, field tests of PQ sensing and analysis in the smart substation by IAD are presented and discussed, including similar short-duration PQs, special steady PQ, multi transient PQ, complex PQ, and irregular transient PQ. Because of the faithful sensing and accurate analysis, the events of these PQs can be discovered. It can be inferred from these tests that the merits of IAD are:

(1) Both time features and frequency features can be sensed faithfully at the same time, and the analysis of the features can be accurate, consequently.
(2) The features have specific physical meaning in time–frequency for the event of PQ revealing.
(3) Prior knowledge of the measured PQ is not necessary.
(4) Nonlinear component interference is not included.

Further, all these merits of IAD belong to the minimization of *u_t_* and *u_f_* at the same time, which solves the influence of the uncertainty principle.

## 6. Conclusions

When facing the challenges of power quality sensing and analysis in a smart substation, this paper explores problems of unfaithful sensing and inaccurate analysis of the exiting methods problem in view of the uncertainty principle, and proposes a new method, ideal atomic decomposition, to solve the problems. The necessity of minimization of time uncertainty and frequency uncertainty simultaneously is highlighted, and is realized by the new method. By the tests of the various simulated and field power quality signals, the universality and accuracy of the new method are validated. Further, the events of the power qualities can be inferred based on the faithful sensing and accurate analysis of power quality. The intelligence of a smart substation can be enhanced by the new method in the aspect of power quality monitoring.

## Figures and Tables

**Table 1:** Related power quality (PQ) sensing methods analysis.

Method Class	Representative Methods	Advantage	Disadvantage
Non-parameter estimation: Linear methods	S transform [10,11], Wavelet transform [12], Gabor transform [13].	(1) Both time features and frequency features can be sensed. (2) Features have specific physical meaning. (3) Prior knowledge of the measured PQ is not necessary.	(1) Both sensing of time features and frequency features are unfaithful because of the uncertainty principle.
Non-parameter estimation: Non-linear methods	Time–frequency distribution [14].	(1) Both time features and frequency features can be sensed. (2) Prior knowledge of the measured PQ is not necessary. (3) The influence of uncertainty principle is excluded.	(1) Feature sensing is with cross-terms, and can hardly be used along for PQ analysis [13].
Parameter estimation	Kalman filter [15,16], Prony [17].	(1) Both time features and frequency features can be sensed. (2) Features have specific physical meaning. (3) The influence of uncertainty principle is excluded.	(1) Precise prior knowledge of the measured PQ is needed. If the PQ does not comply with the model of parameter estimation, the features sensing is unfaithful.
Singular spectrum analysis	Esprit [18].	(1) The influence of uncertainty principle is excluded.	(1) The features do not have specific physical meaning. (2) Prior knowledge of the measured PQ is needed for the window set of spectrum analysis
Deep learning	Convolutional neural network [19].	(1) Prior knowledge of the measured PQ is not necessary. (2) The influence of uncertainty principle is excluded.	(1) The features do not have specific physical meaning and are not explainable. (2) A large amount of data are needed for the training.

**Table 2:** Results comparison of transient and steady PQ analysis.

	Impulse1			Impulse 2			Harmonics Amplitudes				
	(p.u)	(ms)	(ms)	(p.u)	(ms)	(ms)	(p.u)				
	*b*	*t_k_*	Duration	*b*	*t_k_*	Duration	1st	3rd	5th	7th	9th
True	−0.5	35	3.3	1	110	1	1	0.33	0.2	0.14	0.11
IAD	−0.503	35	3.2	1.012	110	1	1.001	0.330	0.201	0.139	0.112
ST1	0.085	33	3.8	0.18	107	3	1.005	0.331	-	-	-
ST2	0.031	33	11	0.06	105	10	1.001	0.330	0.181	0.129	0.100

**Table 3:** Results comparison of voltage sag analysis.

Voltage Sag	Sag Depth (p.u)	Start Time (ms)	Duration (ms)
True	0.5	65	45
IAD	0.5	63	48
DWT1	0.44	63	48
DWT2	0.49	70	49

## References

[1] Huang Q., Jing S., Li J., Cai D., Wu J., Zhen W. (2017). Smart substation: State of the art and future development. IEEE Trans. Power Deliv..

[2] Wang Z., Perera A. (2020). Integrated platform to design robust energy internet. Appl. Energy.

[3] Shahzad Y., Javed H., Farman H., Ahmad J., Jan B., Zubair M. (2020). Internet of energy: Opportunities, applications, architectures and challenges in smart industries. Comput. Electr. Eng..

[4] Chan J.Y., Milanovic J.V., Delahunty A. (2009). Generic failure-risk assessment of industrial processes due to voltage sags. IEEE Trans. Power Syst..

[5] Zhang W., Xiao X., Kai Z., Xu W., Jing Y. (2017). Multicycle incipient fault detection and location for medium voltage underground cable. IEEE Trans. Power Deliv..

[6] Ghandehari R., Shoulaie A. (2009). Evaluating voltage notch problems arising from AC/DC converter operation. IEEE Trans. Power Elec..

[7] Joon-Ho K., Jin O.K. (2020). Analysis and mitigation on switching transients of medium-voltage low-harmonic filter banks. Energies.

[8] The Institute of Electrical and Electronics Engineers, Inc. (2009). Standard IEEE 1159-2009: IEEE Recommended Practice for Monitoring Electric Power Quality.

[9] International Electrotechnical Commission (2003). Standard IEC 61000-4-30:2003: Electromagnetic Compatibility (EMC)–Part 4–30: Testing and Measurement Techniques–Power Quality Measurement Methods.

[10] He S., Li K., Zhang M. (2013). A real-time power quality disturbances classification using hybrid method based on S-transform and dynamics. IEEE Trans. Instrum. Meas..

[11] Beuter C., Oleskovicz M. (2020). S-transform: From main concepts to some power quality applications. IET Signal Proces..

[12] Lin C., Wang C. (2006). Adaptive wavelet networks for power-quality detection and discrimination in a power system. IEEE Trans. Power Deliv..

[13] Cho H., Jang G., Kwon H. (2010). Time-frequency analysis of power-quality disturbances via the Gabor-Wigner transform. IEEE Trans. Power Deliv..

[14] Wright P. (1999). Short-time Fourier transforms and Wigner-Ville distributions applied to the calibration of power frequency harmonic analyzers. IEEE Trans. Instrum. Meas..

[15] Chen C., Chang G., Hong R., Li H. (2010). Extended real model of Kalman filter for time-varying harmonics estimation. IEEE Trans. Power Deliv..

[16] He S., Li K., Zhang M. (2014). A new transient power quality disturbances detection using strong trace filter. IEEE Trans. Instrum. Meas..

[17] Peng J., Nair N. (2009). Adaptive sampling scheme for monitoring oscillations using Prony analysis. IET Gener. Transm. Distrib..

[18] Gu I., Bollen M. (2008). Estimating interharmonics by using sliding-window ESPRIT. IEEE Trans. Power Deliv..

[19] Wang S., Chen H. (2020). A novel deep learning method for the classification of power quality disturbances using deep convolutional neural network. Appl. Energy.

[20] Donoho D., Huo X. (2001). Uncertainty principles and ideal atomic decomposition. IEEE Trans. Inf. Theory.

[21] DeBrunner V., Ozaydın M., Przebinda T. (1999). Resolution in time-frequency. IEEE Trans. Signal Process..

[22] Ślesicka A., Kawalec A. (2020). An application of the orthogonal matching pursuit algorithm in space-time adaptive processing. Sensors.

[23] Stockwell R., Mansinha L., Lowe R. (1999). Localization of the complex spectrum: The S transform. IEEE Trans. Signal Process..

[24] Costa F. (2014). Boundary wavelet coefficients for real-time detection of transients induced by faults and power-quality disturbances. IEEE Trans. Power Deliv..

[25] International Electrotechnical Commission (1997). Standard IEC 61000-4-15. Electromagnetic Compatibility (EMC)–Part 4-15: Testing and Measurement Techniques–Flickermeter-Functional and Design Specification.

[26] The Institute of Electrical and Electronics Engineers, Inc. (1993). Standard IEEE 519-1992. IEEE Recommended Practices and Requirements for Harmonic Control in Electrical Power Systems.

[27] International Electrotechnical Commission (2003). Standard IEC 61850-9-1. Communication Networks and Systems in Substations–Part 9-1: Specific Communication Service Mapping–Sampled Values Over Serial Unidirectional Multidrop Point to Point Link.

[28] Koch B., Christophe P. (1993). Arc voltage for arcing faults on 25(28)-kV cables and splices. IEEE Trans. Power Deliv..

[29] Smith D., Swanson S., Borst J. (1975). Overvoltages with remotely-switched cable-fed grounded wye-wye transformers. IEEE Trans. Power Appar. Syst..

[30] Zhang Y., Yang H., Wang J., Geng Y., Liu Z., Jin L., Yu L. (2016). Influence of high-frequency high-voltage impulse conditioning on back-to-back capacitor bank switching performance of vacuum interrupters. IEEE Trans. Plasma Sci..

[31] National Disturbance Library by IEEE PES. https://pqmon.epri.com/disturbance_library/.

